# Casting a Wider NET: Pancreatic Exocrine Insufficiency Induced by Somatostatin Analogues among Patients with Neuroendocrine Tumours?

**DOI:** 10.3390/cancers15071933

**Published:** 2023-03-23

**Authors:** Lewis A. Hall, Sarah Powell-Brett, Oscar Thompson, Daniel Smith, Elizabeth Bradley, Stacey Smith, Suzanne Vickrage, Joanne Kemp-Blake, Keith J. Roberts, Tahir Shah

**Affiliations:** 1 College of Medical and Dental Sciences, University of Birmingham, Birmingham B15 2TT, UK; 2 The Liver Unit, University Hospitals Birmingham NHS Foundation Trust, Birmingham B15 2TT, UK; 3 Department of Nutrition and Dietetics, University Hospitals Birmingham NHS Foundation Trust, Birmingham B15 2TT, UK; 4 Birmingham Neuroendocrine Tumour Centre, University Hospitals Birmingham NHS Foundation Trust, Birmingham B15 2TT, UKtahir.shah@uhb.nhs.uk (T.S.)

**Keywords:** pancreatic exocrine insufficiency, neuroendocrine tumour, somatostatin-analogues, ^13^C-MTG breath test, faecal-elastase-1

## Abstract

**Simple Summary:**

Somatostatin analogues (SSA) are used to treat patients with unresectable neuroendocrine tumours (NET). An underrecognized side-effect of SSAs is pancreatic exocrine insufficiency (PEI), where the pancreas is unable to maintain the normal digestion of food. Whilst the few papers that have investigated SSA-induced PEI report rates of between 12 and 48%, anecdotal evidence of PEI symptoms suggests this figure is greater. The disparity may be due to the overlap in symptoms of the tumour itself and PEI, but also the diagnostic methods used. Swift diagnosis of PEI is essential to ensure appropriate management of the symptoms for an improved survival and quality of life for patients. This study is the first to utilize the ^13^C-mixed triglyceride breath test (^13^C-MTGT) in patients before and after they commence SSA treatment, demonstrating a novel and alternative diagnostic strategy in this patient cohort, whilst highlighting the early impact of SSAs on pancreatic function.

**Abstract:**

Somatostatin-analogues (SSAs) are a first-line treatment of unresectable neuroendocrine tumours (NETs). However, SSAs inhibit pancreatic secretions, which could lead to pancreatic exocrine insufficiency (PEI). PEI is known to be detrimental to patient quality of life and nutritional status. This study aimed to evaluate the effect of SSAs on pancreatic exocrine function in patients with NETs, using the ^13^C-mixed triglyceride breath test (^13^C-MTGT). Exocrine function was assessed using the ^13^C-MTGT at baseline and after a third SSA injection (two months). A quotient of ^13^CO_2_/^12^CO_2_ was measured by mass spectrometry, and the cumulative percent dose recovered at 6 h (cPDR) is reported. The secondary endpoints investigated were change in weight, HbA1C, and vitamin D levels. Ten patients completed the study. Exocrine function reduced in all patients (n = 10) following SSA therapy (median reduction from baseline: −23.4% (range: −42.1–0.5%, *p* = 0.005)). vitamin D levels decreased in all but one patient (median decrease from baseline: −26.5%, (−44.7–10%; *p* = 0.038)), and median HbA1C levels increased by 8.0% (0–59.3%; *p* = 0.008). Change in weight was not significant (median decrease from baseline: −0.21% (−4.5–3.5%, *p* = 1.000)). SSA therapy has a consistent impact on exocrine function from early in the treatment course, but the long-term clinical effects of this remain to be defined. Further studies are required to determine the clinical relevance of this observation and optimise the management of PEI in this cohort.

## 1. Introduction

Pancreatic exocrine insufficiency (PEI) is a known sequalae of pancreatic pathology leading to maldigestion [1,2]. Prominent symptoms include steatorrhea, bloating, abdominal pain, and flatulence [3,4]. Amongst its more common aetiologies, such as chronic pancreatitis, cystic fibrosis, and pancreatic cancer, PEI is well documented [1,5,6,7,8,9]. However, emerging evidence suggests that PEI may be present in other settings, with poorer recognition [10]. One such setting is in patients with neuroendocrine tumours (NETs) commencing somatostatin-analogue (SSA) therapy. 

Endogenous somatostatin plays a key role in the delicate physiology of the exocrine pancreas. Its release acts via a negative feedback mechanism to prevent continued pancreatic secretion and auto digestion [11]. Exogenous somatostatin acts in a similar fashion, suppressing pancreatic secretion and potentiating the development of PEI [12].

SSA-related PEI in NET patients has a reported incidence of approximately 20% (ranging from 12–48%) in the current literature [12,13,14,15,16]. However, this is thought to be an underestimate, with numbers appearing to be higher in clinical practice [17]. PEI is associated with a poorer quality of life (QoL) and malnutrition; thus, early recognition is encouraged in order to ensure swift management with pancreatic enzyme replacement therapy (PERT) [4,18]. However, several factors hinder an efficient, definitive diagnosis of PEI in this cohort. Foremost, the clinical picture of PEI is ambiguous with a significant overlap between other NET-related symptoms and SSA adverse effects [4,7,10]. Furthermore, the optimum diagnostic strategy for PEI is contentious and, despite various tests available, a paucity of evidence exists regarding the most appropriate test, particularly in the NET cohort [17,19]. The ^13^C-mixed triglyceride breath test (^13^C-MTGT) is a safe, accurate, non-invasive diagnostic test for PEI. Unlike faecal elastase (FE-1) it is not affected by diarrhoea, which can be problematic in this cohort and is more reliable in picking up mild PEI [20,21,22].

The aim of this study is to evaluate the effect of SSAs on pancreatic exocrine function in patients with NETs, using the ^13^C-MTGT. The secondary aim is to assess the impact of SSA on nutritional parameters in these patients, acknowledging the potential contribution of PEI. This study is, to the best of the authors’ knowledge, the first study to directly assess the effect of SSA therapy on pancreatic exocrine function in patients with NETs using the ^13^C-MTGT. 

## 2. Materials and Methods

### 2.1. Study Design

This is a prospective, observational, single-centre cohort study designed to determine the impact of SSAs on pancreatic exocrine function in NET patients. Data are reported in accordance with STROBE Guidance [23]. This cohort of patients is part of the wider DETECTION Study that is currently using the ^13^C-MTGT to evaluate PEI in different cohorts of patients. Our cohort included patients with NETs starting treatment with SSAs at the Queen Elizabeth Hospital, Birmingham (QEHB). We prospectively captured consecutive adult patients commencing SSA therapy who were identified for eligibility at the NET MDT meeting. Eligibility was determined and confirmed by medical practitioners with expert knowledge of NETs and SSA therapy. Healthy volunteers were age- and sex-matched to the included patients and used as controls.

### 2.2. Inclusion Criteria

Eligible patients must have had a confirmed diagnosis of NET, be due to commence SSA therapy, be over 18 years of age, and be able to consent for study participation.

### 2.3. Exclusion Criteria 

Patients with a present diagnosis of other pancreatic disease (e.g., chronic pancreatitis), a history of upper-gastrointestinal surgery that may alter pancreatic function (e.g., gastric bypass), already on SSA therapy, or <18 years of age were excluded. 

### 2.4. Consent 

Where participants fulfilled all eligibility criteria, they were approached by a member of the research team via telephone to invite participation. All patients provided written consent prior to beginning the study procedure. Inability or refusal to complete the study procedures or sign informed consent also warranted exclusion.

### 2.5. Variables 

The collected data include baseline characteristics and three groups of variables: (1) quantitative and (2) qualitative assessment of pancreatic exocrine function, and (3) nutritional status. Routinely, patients receive SSA therapy every 4 weeks at our centre, and data collection was purposefully designed to coincide with these appointments for patient amenability. 

### 2.6. Quantitative Assessment of Pancreatic Exocrine Function

Pancreatic function was measured with ^13^C-MTGT [21]. An amount of 250 mg of ^13^C-MTG (^13^C mixed triglyceride) was incorporated into a test meal and ingested early morning after an overnight fast. The patients were instructed to avoid foods naturally rich in ^13^C (sweetcorn, broccoli, and pineapple) in the 48 h preceding the test, and smoking was prohibited from the prior evening. Patients who had been started on PERT due to developing PEI symptoms during the study period were instructed to withhold PERT for 72 h prior to the breath test, to ensure any decrease in pancreatic function was not masked by residual PERT in the digestive tract. During the test period, the patients were instructed to remain sedentary for the duration, with bathroom breaks the extent of physical activity. Breath samples were collected into 10 mL glass tubes (Exetainer, Labco Limited, Lampeter, UK) at baseline, and then at 60 min intervals for 6 h thereafter. The entire test was conducted at baseline (prior to first SSA injection) and at 8 weeks (on the day of the 3rd injection, after administration). A quotient of ^13^CO_2_/^12^CO_2_ was measured by mass spectrometry and the cumulative percent dose recovered at 6 h (cPDR) is reported [24,25]. The overall reduction in exocrine function from before to after the commencement of SSA therapy is reported. Stool samples were also collected to measure FE-1 levels, as the current diagnostic standard [17,19]. 

### 2.7. Qualitative Assessment of Pancreatic Exocrine Function 

The PEI-Q score was used as the qualitative assessment and change in ‘total symptom score’ (TSS) is reported. PEI-Q is a patient-reported outcome measure that quantifies the clinical assessment of PEI [4]. 

### 2.8. Assessment of Nutritional Status 

Body weight (kg), and body mass index (BMI; kg/m^2^) were measured. Serum vitamin D levels (nmol/L) and HbA1C (mmol/mol) were also collected to assess the potential impact of SSAs and PEI on fat-soluble vitamin concentrations and glycaemic index. 

### 2.9. Statistical Analysis 

Descriptive statistics are used for demographic variables. The Shapiro–Wilk test of normality was used, demonstrating a non-Gaussian population; therefore, non-parametric tests were used where appropriate. Continuous data are expressed as a median (interquartile range; IQR). Categorical variables are presented as absolute values and percentages. The Wilcoxon signed-rank test was used to compare paired samples, from before to after SSA therapy, for each domain of the study. A *p*-value of <0.05 was considered significant. The statistical analysis was conducted using SPSS (IBM Corp. Released 2020. IBM SPSS Statistics for Windows, Version 27.0. Armonk, NY, USA: IBM Corp).

### 2.10. Ethical and Regulatory Considerations 

The study received full Research Ethics Committee (REC) approval (IRAS Project ID—271410). The study was conducted in accordance with guidance outlined in the 1975 Declaration of Helsinki and with the conditions and principles of Good Clinical Practice.

## 3. Results

Recruitment was open from January 2021 to October 2021. A total of eleven patients fulfilled inclusion criteria and agreed to study participation. One patient had to withdraw before completion due to failure to tolerate the SSA therapy. Ten patients were ultimately included in the study, consisting of two woman and eight men with a median age of 72 years (63–76). Two patients had pancreatic NETs (without pancreatic duct obstruction), whilst eight were extra-pancreatic (seven small bowel and one lung), and all were well-differentiated on histology. All patients were commencing SSA therapy due to advanced/unresectable disease. Eight patients had lanreotide (Somatuline^®^ Autogel^®^ (Lanreotide autogel) prolonged-release subcutaneous injection) (60 mg) for their first injection and two received octreotide (Sandostatin^®^ LAR^®^ (Octreotide acetate) intramuscular injection) (10 mg), with each increasing their dose to 120 mg and 30 mg, respectively, for their subsequent injections (per hospital protocol). For this study, both will be simply referred to as SSA henceforth. A summary of baseline characteristics is shown in Table 1. 

### 3.1. Quantitative Assessment of Pancreatic Exocrine Function 

**Figure 1:**
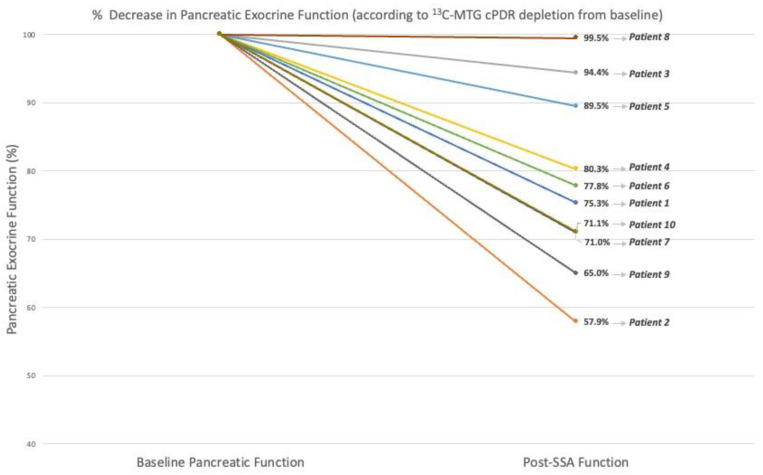
Composite line graph to show change of %cPDR of ^13^C-MTG for each patient.

The ^13^C-MTGT was tolerated in all patients, with no adverse events reported. Six hours of ^13^C-cPDR was significantly reduced from before to after SSA injections commenced, with a median change from baseline of −23.4% (range: −42.1–0.5%, *p* = 0.005). Figure 1 illustrates the change from baseline for each patient. 

Stool samples were obtained from every patient for FE-1 testing at both timepoints. Due to some samples being too liquid to assay, only seven patients had both results available. Levels of FE-1 did not change significantly following SSA therapy with the median change from baseline of 0% (range: −59.5–222.6%; *p* = 0.345) (Table 2).

### 3.2. Qualitative Assessment of Pancreatic Exocrine Function 

**Figure 2:**
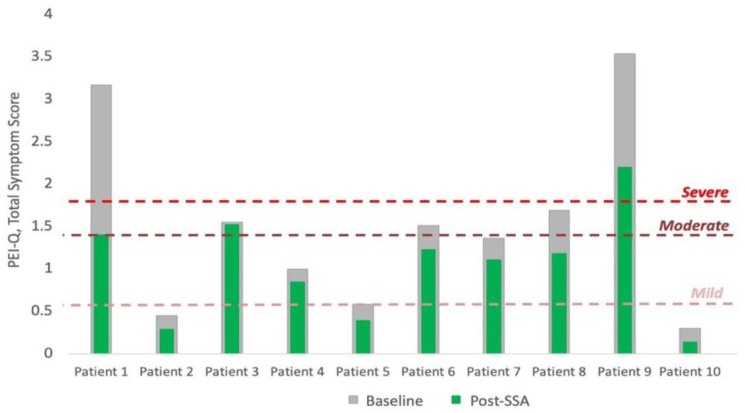
Bar graph of change in total symptom score of PEI-Q, where threshold for ‘mild’, ‘moderate’, and ‘severe’ PEI are at 0.6, 1.4, and 1.8 of total symptom score, respectively. Grey bars represent baseline scores and green bars the scores at 8 weeks (post-SSA).

The PEI-Q was completed by all patients at baseline and after the third injection (2 months). There was a significant decrease (indicating improvement in symptoms) in total symptom score from baseline to after SSA commencement of −31.5% (range: −56.8–1.9%, *p* = 0.005). At baseline, two patients had severe symptom scores, three moderate, two mild, and three reported no PEI symptoms. Seven patients did not have a change in symptom severity after commencing SSA therapy, whilst three patients reported a severity score one class lower than baseline (Figure 2). 

### 3.3. Assessment of Nutritional Status 

**Figure 3:**
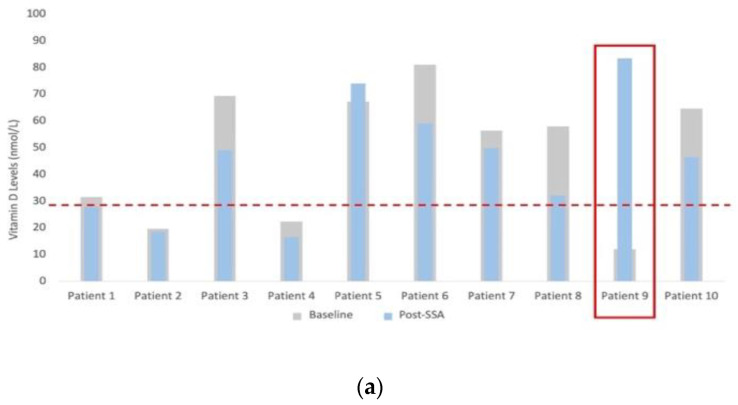

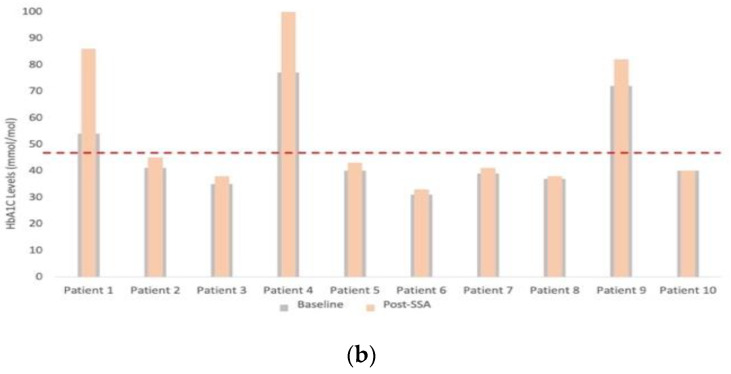
(**a**) Bar graph of change in vitamin D (nmol/L), where a threshold of <29 nmol/L was considered deficient (patient 9 was given vitamin D supplementation between measurements and was thus excluded from the final analysis); (**b**) bar chart of change in HbA1C (mmol/mol), where a threshold of >48 mmol/mol was considered raised. Grey bars represent baseline values.

Vitamin D levels significantly decreased across the cohort, decreasing in eight of nine patients, with a median change from baseline of −26.5%, (−44.7–10%; *p* = 0.038) (Figure 3a). One patient was excluded from the analysis, as they were prescribed vitamin D at the first visit due to severe baseline levels. HbA1C levels significantly increased across the cohort, with nine of ten experiencing an increase. The median change from baseline was 8.0% (0–59.3%; *p* = 0.008) (Figure 3b). There were no significant changes in weight or BMI from before to after commencing SSA therapy, with a median change from baseline of −0.21% (−4.5–3.5%, *p* = 1.000) and 0.27% (−5.2–5.4%, *p* = 0.722), respectively. No patient experienced clinically significant weight loss (defined as a greater than 5% reduction in weight) within this time frame.

### 3.4. PERT Use 

Two of ten patients were commenced on PERT during the study period (patient 8: 49 days from baseline; patient 9: 28 days from baseline) after developing symptoms of PEI and were assessed to have a clinical need for the therapy. One patient entered the study already having a prescription for PERT, following an episode of acute pancreatitis. The decision to include this patient was made with the support of multiple factors: whilst PERT was prescribed, there was no present diagnosis of PEI confirmed by FE-1, and baseline cPDR was compared and equivalent to 10 healthy controls. Furthermore, all analyses were run excluding this patient and no difference to overall change from before to after therapy was made. With the primary objective of the study to assess a change in pancreatic function, this patient was deemed suitable for inclusion. At the time of writing and following completion of the second ^13^C-MTGT, a further five of ten patients have been started on PERT for symptomatic relief. 

## 4. Discussion

This is the first study to establish a before and after picture of the ‘functional exocrine profile’ of patients with NETs commenced on SSA therapy. This is an important topic because (1) untreated pancreatic exocrine insufficiency causes maldigestion and can affect clinical outcomes [1,3], and (2) systemic symptoms of NET disease can be confused with PEI, making diagnosis and response to treatment unclear [12,13]. The key finding of this prospective study was that all patients showed a reduction in pancreatic exocrine function, 8 weeks after commencing SSA therapy, using the ^13^C-MTGT. The impact of SSA for the treatment of NETs on exocrine function is an underrecognized and important side-effect of the therapy. The second major finding was that the ‘disease’-specific QoL tool, PEI-Q, demonstrated an improvement of symptoms of PEI after patients commenced SSA therapy. This is despite clear evidence of a depletion in pancreatic function in this group. The probable reason for this is the symptom overlap between systemic NET disease and that of PEI with the symptom burden of systemic NET disease predominating. Therefore, despite inducing PEI, SSA therapy is associated with an improvement in gastrointestinal QoL measured as the underlying disease is being treated. The relevance of this is that symptoms of PEI cannot be reliably used to suspect, diagnose, or guide therapy in this cohort of patients.

To date, four studies have been published that report the incidence of SSA-related PEI in NETs [12,13,14,16]. The dearth of literature reflects the under-recognition of the condition in this setting. However, understanding the long disease course of the tumours for which SSAs are used to treat, it is clear from the available literature that a significant proportion of patients will develop PEI (11.6–38%), with median time to PEI development between 2.9 and 6.5 months [13,14]. Our results suggest that the depletion in exocrine function may occur earlier than currently reported, as well as more frequently. Why there is such disparity between the current results, and those of the available literature, may be due to differing diagnostic strategies. 

Diagnosing PEI in this population is challenging [17]. There is a significant overlap in symptoms of the tumour and those of PEI. Bloating, urgency, diarrhoea, and steatorrhea may be secondary to the tumour itself, or exocrine insufficiency, and relying on symptomatic assessment alone will underestimate exocrine insufficiency and leave many undertreated [1,4,17]. A specific diagnostic test to identify PEI would mitigate this issue; however, the choice of such a test is not straightforward. 

There is little consensus on the optimum diagnostic test for PEI in any setting, and less still for this aetiology [1,17,19]. Despite the relative inaccuracy of the method, the symptomatic diagnosis of PEI is a common strategy in other settings [1,17]. This strategy is limited in NET patients by the symptom overlap. Furthermore, if the therapeutic management of PEI is to be implemented in this setting, clinicians will want objective evidence to support prescription. FE-1 is widely considered an acceptable diagnostic strategy in most cohorts [17,19], and coupled with its accessibility in most centres, it is typically used to aid diagnoses of PEI in the presence of symptoms, rather than relying on a symptomatic diagnosis alone. Regrettably, its use in the NET setting is limited. Stool analysis requires that the sample be solid enough for testing, and in a population where diarrhoea is so prevalent, it is understandable why five of twenty (25%) samples in the present study could not be analysed. Furthermore, FE-1 is less effective at diagnosing mild PEI [20]. The ^13^C-MTGT allows a specific profile of pancreatic exocrine function, offering early recognition of a decline in pancreatic function, before an official diagnosis, where patients may be less symptomatic [10]. The present cohort demonstrates such, with a median drop in exocrine function of −23.4% (−42.1–0.5) (*p* = 0.005) and all ten patients experiencing a drop from before to during SSA therapy. This may explain the difference between the present findings and the current literature: as the first use of the breath test in this setting, these results corroborate the findings of the available literature, in that SSAs will indeed induce an exocrine insufficiency; however, they further indicate that the impact on pancreatic function occurs very early in the treatment course. The relationship of such dysfunction to patient reported outcomes is paramount, particularly in this unique cohort. 

The results of the PEI-Q questionnaire reiterate the need for the quantitative assessment of exocrine function in this population. The apparent reduction in PEI symptoms after commencing SSAs (−31.5% (−56.8–−1.9) in total symptom score, *p* = 0.005), despite the objective depletion in exocrine function, demonstrates the potential risks of relying solely on symptoms for diagnosing PEI. Whilst PERT prescription in two of ten patients during the study period may have contributed to the improvement in PEI-Q score in these patients, the unanimous improvement in symptom score likely still represents the symptomatic benefit of the SSA, rather than any improvement in PEI symptoms. The disparity in the two domains of assessment is reflected in other studies; Sudeep et al. [26] showed a patient reported symptom rate of 31% vs a CFA-diagnosed rate of 69%. An explanation for such disparity is that symptoms may be masked by other factors, including low-fat diets or constipation. A further benefit of the ^13^C-MTGT and its specificity for pancreatic dysfunction in these patients is its potential to enhance the managing clinicians’ understanding of patients’ symptom aetiology. This is particularly important when considering the potential for the misattribution of refractory symptoms to either worsening carcinoid syndrome or the failure of SSA therapy, rather than the progression of exocrine dysfunction, which could be addressed with an appropriate PERT prescription [13].

PEI is known to negatively affect patients’ QoL [4,27,28] nutritional status and outcomes; thus, early recognition and prompt commencement of PERT is desired. Vitamin D deficiency can lead to metabolic bone disease and osteomalacia [6], so swift recognition and management are essential. The current literature hosts a range of deficiency rates (31–81%) [29,30,31,32,33,34]. This present cohort demonstrated a median drop of 26.5% (*p* = 0.038) in vitamin D levels after starting SSA therapy. Depletion in vitamin D levels may be a direct result of the SSA therapy, or an indirect result through fat-malabsorption, secondary to reduced exocrine function. A further consideration is that there may be seasonal variation in the vitamin D levels of the included patients; the decrease in vitamin D levels in patients recruited in October, for example, may have been affected by seasonal variation. Future studies may choose to measure all fat-soluble vitamins to mitigate this effect. The supplementation of fat-soluble vitamins is an important consideration for clinicians managing patients on SSAs, in order to optimise the nutritional status of the patients and mitigate any consequences [35].

Emerging work is highlighting the complexities of the exocrine–endocrine interface of the pancreas, and whilst response to SSAs can vary, a significant impairment of glycaemic control can occur [36,37,38]. Such variability may be the consequence of a balance of inhibitory effects on the pancreatic beta cells, leading to reduced insulin secretion, and the reduction in circulating growth hormone and insulin-like growth factor-1, resulting in reduced insulin requirements [38]. Rinzivillo et al. [16] demonstrated HbA1C is associated with an increased risk of PEI development after commencing SSA therapy. The authors demonstrated that an elevated HbA1C level was a significant risk factor for PEI development in a multivariate analysis (OR:4.81, 95%CI: 1.33–17.33; *p* < 0.01). The present results reiterate the impact of SSAs on glycaemic control and highlight the contextual importance of concurrent pancreatic function. The significant increase in HbA1C (median increase of 8%, *p* = 0.008), combined with a significant decrease in exocrine function, may indict poor exocrine function as a contributor to deranged glycaemic control. Whatever the pathogenesis, the clear disruption to the endocrine pancreas warrants input from the diabetic team for patients commencing SSA therapy. 

This study is limited by the small number of participants and its single-centre status. Conducting the study through the second wave of the COVID-19 pandemic restricted recruitment volume. Further studies should look to increase the total number of patients included, perhaps through collaborative means and multi-centre involvement. Another limitation of the study is the reproducibility of the standard ^13^C-MTGT procedure in a routine clinical setting, beyond the support that accompanies a research environment. The authors recognise the difficulty and expense of implementing the ^13^C-MTGT into everyday clinical practice; however, the present results from its use provide further assurance that PEI is an important side-effect of SSA therapy. The combination of results demonstrated in this study supports the conclusions of Panzuto et al. [17] in establishing a diagnostic profile for PEI in patients commencing SSA therapy, comprising serial assessments of: PEI (with the available quantitative test), vitamin D and HbA1C blood tests, patient weight/BMI, and PEI-Q scoring. This work suggests that most patients on SSA will be prescribed PERT eventually, and with retrospective data suggesting a potential survival advantage for patients on PERT [39], symptomatic patients may benefit from an trial of PERT early in the treatment course. A randomised control trial should be completed to objectify the benefits. 

## 5. Conclusions

With no consensus on the optimum diagnostic strategy, a dearth of evidence on PEI management in the setting of SSA therapy and NETs, and the present study demonstrating a consistent impact on exocrine function from early in the treatment course, there is definite need for further work in this area. The apparent symptomatic improvement on commencing SSA therapy should not offer complete reassurance to managing clinicians, and the concomitant effect on vitamin D and HbA1C levels should encourage careful monitoring of these patients as they commence treatment. Future studies should establish long-term outcomes for this patient group, with further focus on anthropometric outcomes, whose change may require a longer follow-up period to become apparent. In determining the long-term clinical impact of PEI in these patients, formal guidance must be developed to optimise the diagnosis and management of PEI in this population.

## Figures and Tables

**Table 1:** Baseline characteristics.

Patient	Patient 1	Patient 2	Patient 3	Patient 4	Patient 5	Patient 6	Patient 7	Patient 8	Patient 9	Patient 10
Age *[years]*	63	58	73	72	78	64	84	62	65	76
Sex *[M/F]*	F	M	M	M	M	M	M	M	F	M
Weight *[kg]*	83.5	100.9	72.6	107	68.8	86.1	79.2	86.4	81.1	70.6
BMI *[kg/m^2^]*	36.62	28.5	24	34.5	23.5	27.1	26.9	28.3	31.7	24.7
SSA Type	L	L	L	O	O	L	L	L	L	L
**Tumour**										
Location	Pancreas	Pancreas	Small Bowel	Small Bowel	Small Bowel	Lung	Small Bowel	Small Bowel	Small Bowel	Small Bowel
Grade	1	1	2	1/2	1	2	NR	1	1	1
Ki-67 *[%]*	<1%	<2%	10–15%	<3%	2%	5%	NR	<1%	<1%	3%
Functional Status *	NFu	NFu	Fu	Fu	Fu	NFu	Fu	NFu	NFu	Fu
Histology	WD	WD	WD	WD	WD	WD	WD	WD	WD	WD

* Functional status of the tumour was determined by baseline symptomatology of the patients and 24 h urinary 5-hydroxyindoleacetic acid (5-HIAA) levels (where applicable). **Abbreviations**: F, female; M, male; BMI, body mass index; SSA, somatostatin analogue; L, lanreotide; O, octreotide; WD, well-differentiated; NR, not recorded; Fu, functioning; NFu, non-functioning

**Table 2:** Before and after SSA functional exocrine profile.

		Baseline	2 Months (3 Injections)	Change (Median, *IQR*)	*p*-Value *
**Quantitative Assessment of Function**	**^13^C-MTG Breath Test** (*% cPDR*)	53.1 (44.6–58.9)	38.7 (35.0–45.0)	−23.4% (−42.1–0.5)	**0.005**
	**FE-1** (*ug/g*)	450 (158.8–500)	500 (428–500)	0% (−59.5–222.6)	0.345
**Qualitative Assessment of Function**	**PEI-Q** (*Total Symptom Score*)	1.4 (0.7–1.7)	1.1 (0.5–1.4)	−31.5% (−56.8–−1.9)	**0.005**
**Nutritional Markers**	**Vitamin D** (*nmol/L*)	57.1 (24.6–66.5)	47.6 (28.9–56.7)	−26.5% (−44.7–10)	**0.038**
	**HbA1C** (*mmol/L*)	40 (37.5–50.8)	42 (38.5–72.8)	8.0% (0–59.3)	**0.008**
	**Weight** (*kg*)	82.3 (74.3–86.3)	82.6 (74.3–87.8)	−0.21% (−4.5–3.5)	1
	**BMI** (*kg/m^2^*)	27.7 (25.3–30.9)	27.2 (25.3–32.2)	0.27% (−5.2–5.4)	0.722

* Wilcoxon signed-rank statistical test used to compare paired data; pre- and post-SSA therapy for the same patient.

## Data Availability

The data that support the findings of this study are available from the corresponding author upon reasonable request.
